# Greasing the wheels of inflammasome formation: regulation of NLRP3 function by S-linked fatty acids

**DOI:** 10.1042/BST20241738

**Published:** 2025-01-21

**Authors:** Daniel M. Williams, Andrew A. Peden

**Affiliations:** School of Biosciences, University of Sheffield, Western Bank, Sheffield, S10 2TN, United Kingdom

**Keywords:** inflammation, NLRP3, inflammasomes, s-acylation, palmitoylation, post-translational modifications, membrane trafficking

## Abstract

NLRP3 is an inflammasome seeding pattern recognition receptor that initiates a pro-inflammatory signalling cascade in response to changes in intracellular homeostasis that are indicative of bacterial infection or tissue damage. Several types of post-translational modification (PTM) have been identified that are added to NLRP3 to regulate its activity. Recent progress has revealed that NLRP3 is subject to a further type of PTM, S-acylation (or palmitoylation), which involves the reversible addition of long-chain fatty acids to target cysteine residues by opposing sets of enzymes. This review provides an overview of recent studies that have identified S-acylation as an important modifier of NLRP3 function. The essential role of S-acylation in the recruitment of NLRP3 to intracellular membranes and the consequences of S-acylation-dependent membrane recruitment on NLRP3 localisation and activation are discussed in detail.

## Introduction

Our innate immune system is the first line of defence against exogenous threats and is composed of a network of cells that play an essential role in sensing, responding to and resolving pathogenic infection and tissue damage. The ability of innate immune cells to respond to perceived threats is dependent on the presence of germ-line encoded pattern recognition receptors (PRR), of which there are several classes [1]. Each PRR recognises the presence of a specific molecular signature of microbial infection (pathogen-associated molecular pattern (PAMP)) or tissue damage (damage-associated molecular pattern (DAMP)) and initiates a signalling cascade that leads to the production and release of molecules that co-ordinate a protective inflammatory response tailored to the threat encountered [1].

A key class of PRR are those that seed the formation of an inflammasome, a specialised reaction centre responsible for the processing and extracellular release of the pro-inflammatory cytokines interleukin-1β (IL-1β) and interleukin-18 (IL-18) [2]. Seven receptors have been described to date that are capable of nucleating an inflammasome, with each receptor activated by either direct binding to specific molecules or through indirect sensing of conditions indicative of perturbed cellular homeostasis [3]. The inflammasome receptor NLRP3 (NACHT, leucine-rich repeat (LRR) and PYD domain containing protein-3) is activated by diverse stimuli that induce a loss of intracellular potassium and trigger a canonical NLRP3 activation pathway [4,5]. These stimuli include, but are not limited to, bacterial ionophores (nigericin, gramicidin) [4-6], agents that damage lysosomal membranes (cholesterol crystals, amyloid β) [7-9] and endogenous molecules released from dying or damaged cells (ATP) [5,6,10]. Although the majority of NLRP3 stimuli are unified by their ability to deplete intracellular potassium, potassium-independent NLRP3 stimuli have also been described [11] alongside alternative pathways of NLRP3 activation [12].

**Figure 1:**
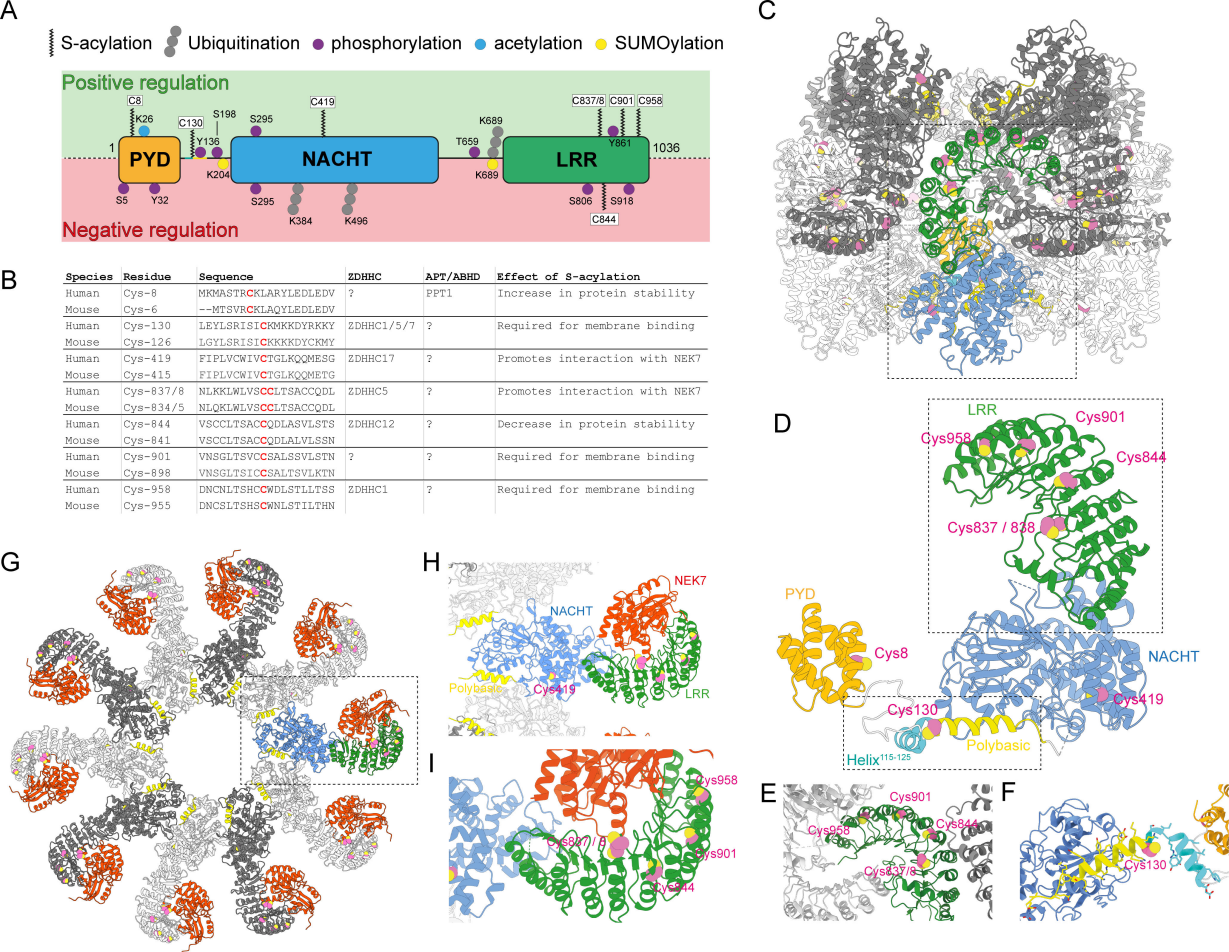
Summary of proposed NLRP3 S-acylation sites and their positions in the active and inactive NLRP3 structures. (**A**) NLRP3 domain architecture showing the position of S-acylation sites identified in NLRP3 to date relative to the position of other types of post-translational modification (PTM) added to NLRP3. A positive or negative regulatory event is defined by the effect of the addition of the indicated PTM on NLRP3 activation, e.g., phosphorylation of Serine5 is negative as phosphomimetic mutations at this position prevent NLRP3 activation [13]. The numbering of each residue is based on its position in the human NLRP3 sequence. (**B**) Table summarising the effect of each S-acylation event on NLRP3 function alongside the machinery involved and the sequence surrounding the modified cysteine residue (coloured in red). (**C**) ChimeraX graphic of the inactive decameric NLRP3 complex from [14]. Pairs of interacting NLRP3 monomers are shown in grey and light grey, with the PYD, NACHT and LRR domains from an individual monomer colour coded (boxed). S-acylated cysteine residues are coloured pink. (**D**) Inactive NLRP3 monomer from boxed region showing the position of each proposed S-acylation site in greater detail. Boxed regions indicate zoom ins of subsequent panels. (**E**) Zoomed region showing the position of proposed S-acylated cysteine residues within the LRR domain (coloured in green). LRR domains from neighbouring NLRP3 monomers that form part of the inactive NLRP3 decamer are shown in grey and light grey. (**F**) Zoomed region showing the position of proposed S-acylated cysteine involved in membrane binding at position 130 between the polybasic domain (coloured in yellow) and helix^115–125^ (coloured in cyan). (**G**) Structure of the active NLRP3 disk bound to NEK7 (coloured in red) from Xia et al. [15]. NLRP3 monomers are coloured grey or light grey with proposed S-acylated cysteine residues in pink. (**H**) Zoomed region showing the position of S-acylated cysteine residues. The NACHT and LRR domains from an individual NLRP3 monomer. Cys-8 and Cys-130 are not shown as residues 1–132 were not resolved in Xiao et al. [15]. (**I**) Detailed view of proposed S-acylated cysteine residues within the LRR domain of an activated NLRP3 monomer.

NLRP3 consists of an N-terminal PYD domain, a central AAA ATPase nucleotide-binding domain (known as the NACHT domain) and a C-terminal LRR domain (Figure 1A). In its inactive state, NLRP3 is bound to ADP and adopts a cage-like structure made up of 10–16 NLRP3 monomers with the PYD domains shielded within the core of this complex [14,16]. In response to NLRP3 stimuli, the NACHT domain exchanges ADP for ATP and undergoes conformational changes that are necessary for NLRP3 activation [14-22]. These rearrangements lead to formation of an oligomeric NLRP3 ‘disk’ [15] that exposes the NLRP3 PYD domain and allows binding to the PYD domain of an adaptor molecule, apoptosis-associated speck-like protein containing a CARD (ASC). This interaction templates the formation of large filamentous ASC assemblies [23-26]. ASC filaments are cross-linked and condensed into a single aggregate that serves as a platform for the concentrated recruitment and activation of the enzyme caspase-1, which in turn converts IL-1β and IL-18 into their active forms [27-29]. Inflammasome activation also leads to a lytic and inflammatory form of cell death, pyroptosis, that allows IL-1β and IL-18 to exit the cell and exert their effects [30].

Pathogenic inflammatory responses can occur when inflammasome receptors are activated in the absence of an injury or infection by endogenous molecules that perturb intracellular homeostasis in a way that matches the signatures of bacterial infection or tissue damage. Engagement of the innate immune system under these circumstances can lead to destructive and self-perpetuating cycles of sterile inflammation that cause or exacerbate disease [31]. This scenario is well described for NLRP3 whose activation in response to a variety of self-derived DAMPs contributes to the pathogenesis of Alzheimer’s, diabetes and atherosclerosis, alongside many other diseases [7-9,32-34]. As a result of its involvement in diseases that represent a significant global health burden, the mechanisms underpinning NLRP3 activation have been the subject of intense study. Among many key discoveries [4,35-37], these efforts have identified that NLRP3 is subject to a wide variety of post-translational modifications (PTMs) that act to keep NLRP3 activation under tight control through diverse mechanisms (Figure 1A) [13,38-44].

Several groups have recently shown that NLRP3 undergoes S-acylation [45-54], a type of reversible PTM that involves the conjugation of fatty acids to cysteine residues. This mini review provides an update on recent work in this area. A brief introduction to protein S-acylation and the cellular machinery involved in this process is first provided prior to a description of studies that have identified S-acylation as an important regulator of NLRP3 function.

## Background to protein S-acylation

S-acylation of proteins involves the attachment of long-chain fatty acids of varying lengths (most often palmitate, C16:0) to specific cysteine residues on target proteins, with current estimates suggesting that between 10% and 20% of human proteins undergo S-acylation [55]. This modification in turn influences protein behaviour, as discussed further below. In contrast with other types of protein lipidation such as myristoylation, geranylgeranylation and farnesylation, protein S-acylation can be rapidly reversed through the actions of enzymes with opposing functions. The addition of S-acyl chains to target cysteine residues on a protein is catalysed by a family of multipass integral membrane proteins that are (1) zinc-dependent [56,57], (2) contain a characteristic DHHC motif as part of their active site and (3) utilise Acyl-CoA as their substrate. In mammals, there are up to 23 ZDHHC enzymes (also known as protein acyltransferases (PATs)) that localise throughout the secretory and endocytic pathway [58-60] and S-acylate distinct substrates [61,62]. The catalytic mechanism of protein S-acylation first involves auto-acylation of the ZDHHC enzyme itself with the head of the acyl chain covalently bound to the catalytic cysteine and the tail held within a membrane-embedded binding pocket formed by the transmembrane domains of the enzyme [63,64]. Upon encounter with a protein containing a cysteine residue amenable to S-acylation, the acyl-chain held by the enzyme is transferred to the sulphur atom of the target cysteine residue and covalently attached through a thioester linkage [63-66]. Since the Acyl-CoA donor utilised by ZDHHCs remains within the cytosolic leaflet of the membrane bilayer throughout this process, S-acylation of both cytosolic and transmembrane proteins typically results in tight association of the modified region with the membrane.

Removal of S-linked acyl chains from cysteine residues is catalysed by the APT (acyl-protein thioesterase) and ABHD (alpha beta hydrolase domain) family of thioesterase enzymes [67,68]. APT and ABHD proteins each contain an ABH domain that forms the active site of the enzyme. In contrast with the ZDHHC enzymes, thioesterases are predominantly peripheral membrane proteins that are themselves recruited to intracellular membranes in a reversible manner through S-acylation by ZDHHC proteins [69-72]. Each thioesterase enzyme also displays a characteristic localisation that probably helps to determine the substrates they act on. For instance, APT1 is present on mitochondria [71], APT2 is found on the Golgi [70] and ABHD17A is enriched at the plasma membrane [72,73]. De-acylation is proposed to occur in two steps [67,70]. Collisions between S-acylated proteins and thioesterases on the organelle surface first lead to extraction of cysteine-linked acyl chains from the membrane bilayer and insertion into the active site of the enzyme [70]. Catalytic residues within the ABH domain then cleave the thioester linkage between cysteine and acyl chain, ‘de-acylating’ S-acylated proteins. The reversible nature of S-acylation therefore means that any effect of S-acylation on protein function can be turned off and on, often in response to specific stimuli [74-76].

S-acylation of proteins can regulate protein function in multiple ways, with perhaps the best known being the reversible recruitment of otherwise soluble cytosolic proteins to intracellular membranes [77-79]. However, S-acylation can also facilitate protein-protein interactions, influence protein stability and induce conformational changes necessary for protein function [74]. To date, all four of these outcomes have been proposed to occur following S-acylation of NLRP3 at distinct cysteine residues. Studies that have identified S-acylation as an important regulator of NLRP3 are described below and are grouped by the effect that each individual S-acylation event is proposed to have on NLRP3 function (Figure 1A and B).

## Regulation of NLRP3 stability by S-acylation

Two groups have found that S-acylation of NLRP3 regulates its stability [45,54]. NLRP3 was shown to be S-acylated within the LRR domain at position 844 (Cys-841 in mouse NLRP3) by the Golgi localised PAT ZDHHC12 [45]. S-acylation at Cys-844 by ZDHHC12 promotes NLRP3 degradation post-activation to attenuate NLRP3 signalling; knockout of ZDHHC12 in macrophages elevated total NLRP3 protein levels and enhanced the amount of IL-1β released in response to NLRP3 activators both in cell culture and *in vivo*. Control of NLRP3 levels through S-acylation at Cys-844 was proposed to occur through recognition of KFERQ motifs present in NLRP3 by Hsc70 (heat shock cognate protein of 70 kDa) and routing of NLRP3 for degradation through chaperone-mediated autophagy (CMA). The authors speculated that S-acylation at Cys-844 by ZDHHC12 induces a conformational change in NLRP3 to expose a KFERQ motif at positions 798–802 and allow binding to Hsc70. Further work has demonstrated that ABHD8 facilitates ZDHHC12-mediated clearance of NLRP3 by acting as a scaffold to recruit ZDHHC12, with knockout of ABHD8 phenocopying loss of ZDHHC12 function [53]. Cys-844 is not surface exposed in the inactive NLRP3 complex (Figure 1C–E) suggesting that NLRP3 decamers may be protected from Cys-844-induced degradation. Conformational changes involved in transition of NLRP3 to its active state that make Cys-844 more accessible may therefore be essential for efficient S-acylation of this residue and removal of active NLRP3 monomers or disks by CMA (Figure 1G–I).

A second report has identified Cys-8 (Cys-6 in mice) as a separate residue that undergoes S-acylation to regulate NLRP3 stability [54]. Through investigation of the regulatory mechanisms driving excessive NLRP3-dependent inflammation in diabetic foot ulcers, the authors discovered that the thioesterase palmitoyl-protein thioesterase 1 (PPT1) regulates NLRP3 stability through de-acylation of NLRP3 at Cys-8. PPT1 knockdown increased both total NLRP3 protein levels and levels of NLRP3 S-acylation with the effect of PPT1 knockdown on NLRP3 lost when Cys-8 was mutated. The action of PPT1 on NLRP3 to remove an acyl-chain added at Cys-8 therefore serves to destabilise NLRP3 protein levels. However, the enzyme responsible for S-acylation and stabilisation of NLRP3 at Cys-8 is not known nor is how S-acylation at Cys-8 may stabilise NLRP3. Where in the cell PPT1 acts on NLRP3 is also not fully understood. As PPT1 is thought to predominantly act on substrates within the lysosomal lumen to remove acyl chains from proteins targeted for degradation [80-83], NLRP3 may be de-acylated by PPT1 within the lysosomal lumen.

## S-acylation-dependent regulation of NEK7 binding

Two S-acylation sites in NLRP3 have been identified that are proposed to be important for the interaction of NLRP3 with NEK7, an essential NLRP3 binding partner [35,84]. Zheng et al. found that NLRP3 is S-acylated by ZDHHC5 at residues 837 and 838 within the LRR domain (Figure 1C and D) [46]. In contrast with the inhibitory effect of Cys-844 on NLRP3 signalling post-activation, S-acylation at Cys-837/838 promotes inflammasome formation, with mutations in either of these residues blocking NLRP3 activation. Overexpression of a ZDHHC enzyme library identified ZDHHC2, ZDHHC3, ZDHHC5 and ZDHHC7 as PATs capable of enhancing NLRP3 S-acylation. However, in this study, only knockdown of ZDHHC5 had any impact on basal levels of NLRP3 S-acylation, implying that ZDHHC5 is the major NLRP3 S-acyltransferase. Knockout of ZDHHC5 limited ASC speck formation in mouse macrophages and resulted in reduced levels of circulating IL1β in NLRP3-dependent models of inflammation *in vivo*, demonstrating that ZDHHC5 positively regulates NLRP3 activation via S-acylation. As mutation of Cys-837 or Cys-838 reduced the amount of NLRP3 pulled down by NEK7, ZDHHC5 was proposed to S-acylate NLRP3 at Cys-837/838 and promote NLRP3 activation by facilitating its interaction with NEK7, potentially through binding of the acyl chains added to Cys-837/Cys-838 to a hydrophobic cleft on NEK7. While Cys-837/838 are in close proximity to NEK7 in the active NLRP3 disk (Figure 1G–I), these residues are buried within the core of the inactive complex. Thus, as for Cys-844, structural rearrangements that accompany NLRP3 activation may again be necessary for S-acylation of Cys-837/8. However, whether acyl groups added to Cys-837/838 directly interact with NEK7 will need further validation as this proposed mode of interaction would require that the acyl chains added to Cys-837/838 are first extracted from the membrane, although how this may be achieved is not known.

In a second study, S-acylation of NLRP3 at Cys-419 within the NACHT domain was proposed to be important for NLRP3 activation by allowing it to interact with NEK7 [52]. In NLRP3 knockout cells expressing NLRP3^C419S^, the interaction of NLRP3 with NEK7, ASC oligomerisation and IL1β secretion were all reduced in response to NLRP3 stimuli, demonstrating that Cys-419 is a functionally important residue. These alterations in NLRP3 functionality corresponded with a reduction in levels of NLRP3^C419S^ S-acylation. Through overexpression of a ZDHHC enzyme library in HEK-293T cells, ZDHHC17 was the only enzyme identified in this study that was capable of enhancing NLRP3 S-acylation levels, leading the authors to propose a model whereby ZDHHC17 S-acylates NLRP3 at Cys-419 to promote the interaction with NEK7.

## S-acylation-dependent membrane recruitment of NLRP3

Work by multiple labs has demonstrated that S-acylation also regulates the association of NLRP3 with intracellular membranes. Four groups have identified Cys-130 (Cys-126 in mouse) as being important for recruitment of NLRP3 to the Golgi (Figure 1C–F) [47-50]. This modification is essential for NLRP3 function as mutation of Cys-130 significantly impairs NLRP3 activation [47,49,50]. Several PATs have been identified that have a significant impact on both total NLRP3 S-acylation levels and NLRP3 localisation through Cys-130. When overexpressed, ZDHHC3, ZDHHC5 and ZDHHC7 all lead to a Cys-130-dependent increase in NLRP3 S-acylation when visualised biochemically using acyl-exchange assays [47-49] and enhance levels of NLRP3 at the Golgi (ZDHHC3, ZDHHC7) or plasma membrane (ZDHHC5) when visualised by fluorescence microscopy [48]. However, of these PATs, only ZDHHC7 appears to be responsible for Cys-130-dependent recruitment of NLRP3 to the Golgi in macrophages. In contrast with the related enzyme ZDHHC3, whose knockdown had no impact on NLRP3 activation [47], loss of ZDHHC7 function reduced NLRP3 S-acylation levels, abolished recruitment to the Golgi and suppressed NLRP3 activation [47]. Thus, despite the high level of sequence similarity between ZDHHC3 and ZDHHC7 (ZDHHC3 = 299 amino acids, ZDHHC7 = 308 amino acids, 58.9% identity, 72.6% similarity) and the ability of overexpressed ZDHHC3 to recruit NLRP3 to the Golgi at similar levels to ZDHHC7 [48], Cys-130 dependent enrichment of NLRP3 on the Golgi is regulated by ZDHHC7.

Other work has proposed that the ER localised PAT ZDHHC1 is primarily responsible for S-acylation of NLRP3 at Cys-130 [50], although knockdown of ZDHHC7 in this study also reduced recruitment of NLRP3 to the Golgi. A fraction of NLRP3 may also be recruited to the plasma membrane through S-acylation at Cys-130 by plasma membrane localised PATs such as ZDHHC5 [48,49]. Consistent with this, a pool of NLRP3 remains on the PM and tubular recycling endosomes in HeLa cells treated with BrefeldinA [48], demonstrating that PM recruitment of NLRP3 can occur independently of Golgi localised PATs. In addition, treatment of cells with disulfiram, which covalently binds to Cys-130 and exclusively blocks NLRP3 S-acylation at this residue, reduces NLRP3 S-acylation levels in ZDHHC7 knockdown cells [49], indicating that PATs other than ZDHHC7 contribute to Cys-130 S-acylation. As described for Gα proteins [59] and SNAP25 [85], differentially localised PATs could therefore compete to S-acylate a single residue (Cys-130 in this case) to determine the amount of NLRP3 recruited to different organelles. Whether this is indeed the case and what the functional relevance of distinct organelle pools of NLRP3 may be will require further investigation. The relative contributions of each PAT to S-acylation at a particular cysteine are also potentially subject to change under different conditions, with recent work describing an increase in levels of ZDHHC5 in response to mitochondrial ROS induced by NLRP3 stimuli [86].

In contrast with our knowledge of the PATs involved in S-acylation at Cys-130, the thioesterases involved in removal of the acyl chain at Cys-130 have not yet been definitively identified. De-acylation of Cys-130 probably does occur, as inhibition of thioesterase activity using PalmostatinB leads to re-localisation of NLRP3 to other intracellular membranes downstream of the Golgi [48]. In addition, the pool of NLRP3 on the Golgi can be removed within 15 min using mitochondrial re-routing assays, indicating that all S-acylated NLRP3 on the Golgi is released from the membrane within this timeframe [48]. Candidates involved in de-acylation at Cys-130 include the plasma membrane localised thioesterase ABHD17A, whose knockout elevates NLRP3 S-acylation levels [50], and Golgi localised APT2, whose overexpression limits the amount of NLRP3 on the Golgi [48] and reduces levels of NLRP3 S-acylation [50]. Both APT1 and PPT1 also reduce total NLRP3 S-acylation levels when overexpressed [50]. However, APT1 overexpression has no impact on the amount of NLRP3 recruited to the Golgi [48], suggesting that APT1 may act on residues other than Cys-130. The effect of overexpressed PPT1 on NLRP3 may be due to de-acylation of Cys-8, as described above [54].

Cys-130 may not be S-acylated in the active NLRP3 disk. In the cryo-EM structure of the active NLRP3 complex, nucleation of ASC filaments by the NLRP3 PYD domain occurs on top of the linker region containing the polybasic domain and Cys-130 [15]. In this configuration (Figure 1G and H), the direction of filament growth from the NLRP3 disk does not look to be compatible with membrane association via Cys-130 lipidation, which would orient the disk with its PYD domains against the membrane. This contrasts with the orientation of the polybasic (PB) domain and Cys-130 in the inactive complex [14,16], where the positioning of this region would appear to allow Cys-130-dependent membrane tethering without imposing any steric clashes that could inhibit formation of the inactive NLRP3 complex (Figure 1C–D and F).

Two further residues, Cys-901 and Cys-958, have also been implicated in recruitment of NLRP3 to intracellular membranes. For Cys-958 (Cys-955 in mice), the authors proposed that S-acylation at this residue by ZDHHC1 facilitates interactions between the LRR domains of individual NLRP3 monomers, promoting formation of NLRP3 multimers and association of NLRP3 with the Golgi and mitochondria [50]. The positioning of this residue in the inactive NLRP3 complex is consistent with a potential role in stabilising the decamer (Figure 1C and E). However, as for Cys-837/838, this would again require that the acyl chain attached to Cys-958 be extracted from the membrane. A further study has shown that Cys-901 (Cys-898 in mice) contributes to recruitment of NLRP3 to the Golgi or endosomes as mutation of Cys-901 reduced levels of NLRP3 S-acylation and co-localisation of NLRP3 with TGN46 [51]. As deletion of the LRR domain or mutation of specific residues required for decamer formation has previously been shown to impact both the amount of NLRP3 recruited to the Golgi and NLRP3 activation [16,38,48,87,88], these effects could in part be attributable to the absence of acyl chains added to the LRR domain at Cys-901 or Cys-958.

## A revised model of NLRP3 membrane binding

**Figure 2:**
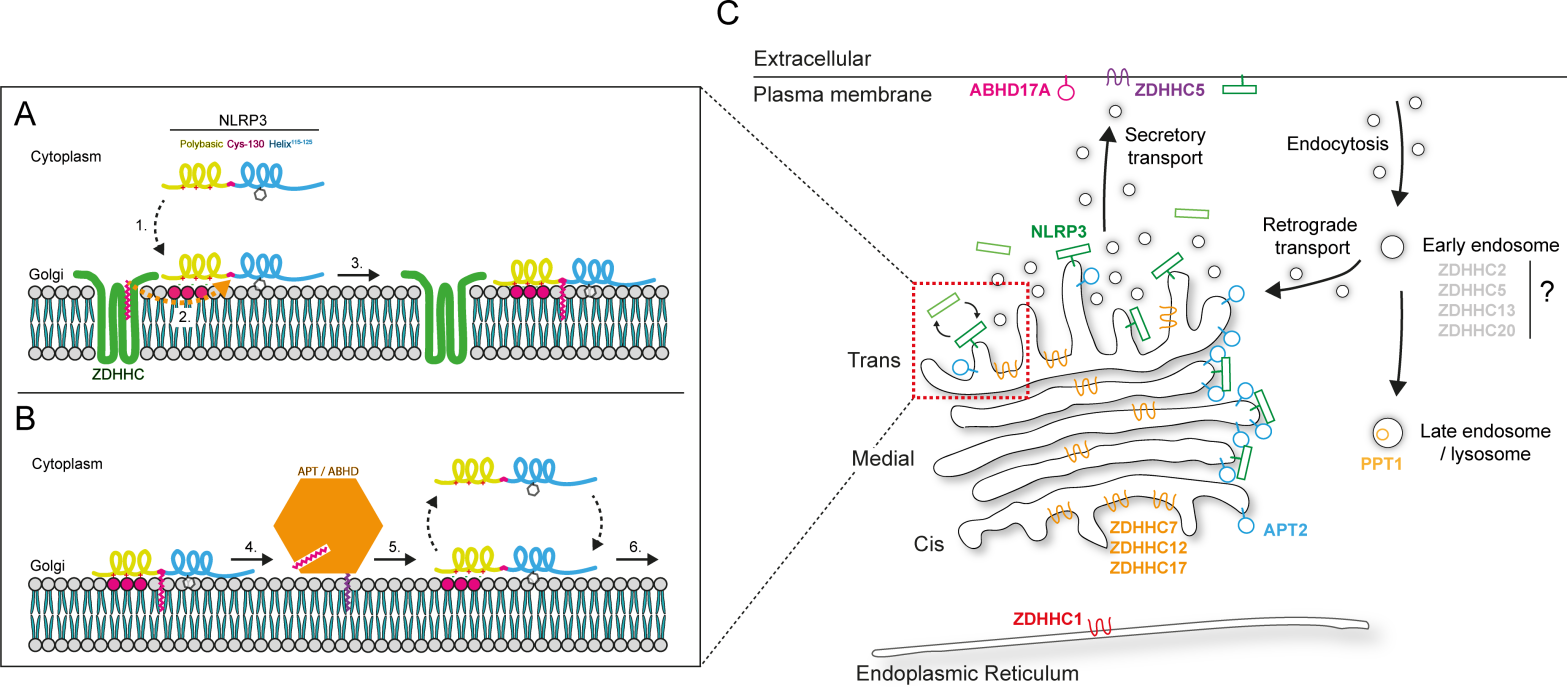
Overview of the proposed NLRP3 membrane binding cycle and localisation of the machinery involved. (**A**) (1) Cytoplasmic non-S-acylated NLRP3 can transiently associate with intracellular membranes through the polybasic (PB) region (coloured in yellow) and helix^115–125^ (coloured in blue). For simplicity, only the PB region, Cys-130 and helix^115–125^ are shown. Negatively charged phospholipids are indicated by magenta headgroups. (2) Non-S-acylated NLRP3 can either dissociate from the membrane or undergo S-acylation (coloured in magenta) at Cys-130 upon encounter with a ZDHHC enzyme. (3) S-acylation of NLRP3 at Cys-130 allows NLRP3 to become stably membrane associated. (**B**) (4) NLRP3 remains stably bound to the membrane until encounter with a thioesterase enzyme, which cleaves the S-acyl chain from Cys-130. (5) Removal of the acyl chain releases NLRP3 from the membrane where it again cycles between a cytoplasmic non-acylated state and a membrane-bound non-acylated state. (6) Membrane-bound non-acylated NLRP3 can re-enter the S-acylation cycle again upon encounter with ZDHHC enzymes. (**C**) Diagram showing the proposed localisation of ZDHHC and APT/ABHD enzymes involved in NLRP3 S-acylation. ER-localised ZDHHC1 (red), plasma membrane–localised ZDHHC5 (purple) and Golgi-localised ZDHHC7 (orange) have all been proposed to modify NLRP3 at Cys-130, suggesting that multiple PATs can S-acylate NLRP3 at the same residue to determine where NLRP3 localises to. De-acylation of Cys-130 on the Golgi could be catalysed by Golgi localised APT2 (blue). NLRP3 could also be de-acylated at the plasma membrane by ABHD17A (magenta) following transport of NLRP3 to this compartment on secretory carriers or following direct S-acylation at Cys-130 and trapping by PM localised PATs such as ZDHHC5. ZDHHC12 and ZDHHC17 (orange), proposed to S-acylate NLRP3 at Cys-844 and Cys-419 to regulate NLRP3 stability and the interaction with NEK7, respectively, are also both known to localise to the Golgi apparatus [58-60]. As NLRP3 has been reported to localise to endosomal membranes [90,91], potential PATs that could S-acylate NLRP3 at this compartment (coloured grey) include ZDHHC2 and ZDHHC5, which both partially localise within the endocytic system [92,93], and ZDHHC13 and ZDHHC20, which may accumulate on endosomes in response to NLRP3 stimuli [94]. PPT1 (yellow) is proposed to de-acylate NLRP3 at Cys-8 and is known to localise to the lysosomal lumen [81,82].

Recruitment of NLRP3 to Golgi or endosomal membranes was originally proposed to occur through electrostatic interactions between the PB domain between residues 131 and 145 in NLRP3 and negatively charged lipids present on organelle membranes [89]. With the identification of Cys-130 as a critical regulator of NLRP3 membrane association, this model can now be updated. While the interaction of the PB region with negatively charged lipids is important [89,90], these interactions cannot enrich NLRP3 on any organelle on their own [48]. Instead, the PB region and hydrophobic residues in a preceding alpha helix (helix^115–125^) probably act together to drive an initial labile mode of membrane binding involving weak electrostatic and hydrophobic interactions. This mode of binding brings NLRP3 in proximity of acyltransferases such as ZDHHC7 that catalyse S-acylation at Cys-130 to stably tether NLRP3 to the cytoplasmic leaflet of the membrane bilayer (Figure 2A). The kinetics of NLRP3 membrane binding via helix^115–125^ and the PB region prior to S-acylation are therefore fundamentally different from those provided by S-acylation of NLRP3 at Cys-130. For NLRP3 molecules that are tethered to the bilayer through S-acylation at Cys-130, Golgi dissociation is much slower, as it requires either encounter with thioesterase enzymes to remove the lipid anchor (Figure 2B) or exit from the Golgi by loading onto secretory transport vesicles. This slower rate of dissociation is likely the reason that NLRP3 shows enrichment on Golgi membranes. Golgi enrichment is lost when either the PB region or helix^115–125^ are mutated, due to NLRP3 having reduced contact with membranes containing ZDHHCs, or when Cys-130 is mutated, as any membrane contact cannot result in S-acylation at Cys-130.

Notably, membrane association via helix^115–125^ and the PB region is not sufficient to fully support NLRP3 activation as NLRP3^C130S^, which retains membrane contact through the PB region and helix^115–125^ but cannot be S-acylated, displays reduced sensitivity to NLRP3 agonists [47,49,50]. Furthermore, deletion of residues 95–134 (NLRP3^Δexon3^), which in theory would significantly limit transient membrane contact, gives a similar phenotype to NLRP3^C130S^ [95]. This would further suggest that the weak membrane binding retained by NLRP3^C130S^ provides no added benefit over a mutant such as NLRP3^Δexon3^ that is likely to be predominantly cytosolic. Optimal sensing of NLRP3 agonists therefore depends on the stable mode of membrane binding provided by S-acylation at Cys-130.

## How does S-acylation-dependent membrane binding support NLRP3 activation?

Relative to other inflammasome seeding PRRs, membrane binding is perhaps one of the defining features of NLRP3s biology. As others have previously proposed [90,96,97], an obvious explanation for the presence of a membrane binding module is that it allows NLRP3 to monitor the health and functionality of the organelle it is bound to. How could S-acylation at Cys-130 support this? S-acylation would confer specific properties, such as longer membrane residence times and the ability to partition into lipid nanodomains [98-104]. These properties may allow NLRP3 to make robust contact with membrane associated proteins or lipids important for its activation. Changes to these properties caused by NLRP3 agonists could also potentially increase the probability of NLRP3 activating. Indeed, alterations in the residence time of NLRP3 on Golgi membranes have been described in response to molecules that perturb Golgi and endosomal function; in HeLa cells, nigericin treatment causes a pool of GFP-NLRP3 to cycle on and off the Golgi with slower kinetics [48]. The Cys-130 S-acylation cycle could therefore act as a molecular timer coupled to the health of the organelle it is bound to, with molecules of NLRP3 that have longer membrane dwell times or altered partitioning behaviour induced by NLRP3 stimuli more likely to activate, potentially due to conformational changes or an increased probability of encountering proteins important for its activation. In agreement with the idea that a stable mode of membrane binding is important for NLRP3 function, an NLRP3 chimera with the PB region replaced by the OSBP-PH domain (Oxysterol-binding protein (OSBP) pleckstrin homology (PH) domain, a high-affinity PI4P binding module that also enriches NLRP3 on the Golgi) was shown to be capable of supporting NLRP3 activation in an *in vitro* assay [89]. However, more recent work found a reduction in the percentage of cells undergoing pyroptosis in NLRP3 KO cells re-expressing this same chimera [16]. S-acylation of NLRP3 at Cys-130 may therefore impart unique biophysical characteristics that are essential for optimal NLRP3 activation.

## Conclusions and future directions

In summary, numerous studies have demonstrated that NLRP3 undergoes S-acylation, which potentially impacts NLRP3 stability, protein-protein interactions and membrane binding. Promisingly, targeting NLRP3 S-acylation represents a potential therapeutic strategy, as NLRP3 activation is inhibited by several drugs that block NLRP3 S-acylation [46,47,49-52]. However, many questions remain regarding the finer details of how S-acylation regulates NLRP3 function. How does NLRP3 interact with so many different PATs, and why is it S-acylated at several distinct residues distributed across its length? Where do these reactions take place, and in what order (Figure 2C)? The dynamics of S-acylation at several sites, and any potential thioesterases involved in their de-acylation, in most cases remains unknown, as does the identity of the PATs involved in S-acylation at Cys-8 and Cys-901. At present, it is also unclear how NLRP3 S-acylation is co-ordinated with other PTMs that NLRP3 is subject to. Given that many NLRP3 PTMs are interdependent [39,44,50], it is reasonable to hypothesise that S-acylation could influence the addition or removal of other PTMs. Finally, due to the way in which S-acylation is commonly measured, it is worth considering that the effect of mutating a specific cysteine residue on NLRP3 S-acylation levels may be indirect. It will therefore be important to provide more direct evidence of S-acylation at a specific residue by mass spectrometry (MS) or other methods. To date, Cys-837/838 are the only residues where S-acylation has been detected by MS [45].

**Perspectives**

- Understanding the regulatory mechanisms of NLRP3 activation is essential to treat diseases driven by aberrant NLRP3 activation. S-acylation is important for NLRP3 function, making targeting of this PTM and the machinery involved an attractive therapeutic strategy.
- NLRP3 is proposed to be S-acylated at multiple residues through distinct enzymes that include ZDHHC1, ZDHHC5, ZDHHC7, ZDHHC12 and ZDHHC17. Modification of each site has a unique impact, with key effects identified so far involving the regulation of NLRP3 stability, facilitation of interaction with NEK7 and recruitment of NLRP3 to intracellular membranes.
- Discrepancies exist between studies regarding the PATs involved in NLRP3 S-acylation and the residues modified. Future work should therefore aim to (1) define a complete set of NLRP3 S-acylation sites, (2) clarify the machinery involved in the addition and removal of acyl chains at each site and (3) obtain an in-depth mechanistic understanding of the impact of S-acylation at each site on NLRP3 function.

## Abbreviations

APT: acyl-protein thioesterase
CMA: chaperone mediated autophagy
DAMP: damage-associated molecular pattern
IL: interleukin
LRR: leucine-rich repeat
MS: mass spectrometry
PAMP: pathogen-associated molecular pattern
PATs: protein acyltransferases
PPT1: palmitoyl-protein thioesterase 1
PRR: pattern recognition receptors
PTM: post-translational modification
